# Influence of Na^+^ disorder on cytoplasmic conductivity and cellular electromagnetic (EM) energy absorption of human erythrocytes (PONE-D-21-36089)

**DOI:** 10.1371/journal.pone.0277044

**Published:** 2023-02-23

**Authors:** Chadapust J. Sudsiri, Raymond J. Ritchie

**Affiliations:** 1 Department of Industrial Management, Faculty of Sciences and Industrial Technology, Prince of Songkla University, Suratthani, Thailand; 2 Biotechnology of Electromechanics Research Unit, Faculty of Technology and Environment, Prince of Songkla University, Kathu, Phuket, Thailand; Shanghai Jiao Tong University, CHINA

## Abstract

Cytoplasmic conductivity of human erythrocytes may be significantly disturbed by the composition of the external suspending media. Effects of external NaCl on cytoplasmic conductivity of human erythrocyte (Human Red Blood Cells, HRBC) were investigated in a simple NaCl system. Using thermodynamic theory cytoplasmic conductivities could be calculated from internal [K^+^], [Na^+^], [Cl^-^] and [HCO_3_^-^]. Effect of cell volume and cell water changes were introduced and allowed for using the Debye-Hückel-Onsager relation and Walden’s rule of viscosity. Cell volume and cell water change of HRBCs were measured in suspending isotonic solutions with conductivities from 0.50 S m^-1^ up to hypertonic solutions of conductivity of 2.02 S m^-1^ at selected temperatures of 25°C (standard benchmark temperature) and 37°C (physiological temperature). In isotonic solutions, cytoplasmic conductivity of human erythrocyte decreases with rise in the external media ionic concentration and *vice versa* for hypertonic solutions. The HRBC is capable of rapidly regulating its volume (and shape) over quite a wide range of osmolality. Specific Absorption Rate (SAR, 900 MHz) values (W kg^-1^) of electromagnetic radiation are below safe limits at non-physiological 25°C but above legal limits at 37°C [National Council on Radiation Protection and Measurements, NCRP]. However, at 37°C under *both* hypertonic [Na^+^] and isotonic but low [Na^+^], SAR increases further beyond legal limits.

## 1. Introduction

An increase in modern electromagnetic technology offers indispensable tools for economic growth and the development of medical equipment. However, technological progress in general has always been associated with both benefits and sometimes unanticipated hazards. Electromagnetic fields (EMF) are composed of the electric field (EF) and the magnetic field (MF). EMFs have become a driving force for human civilization through their numerous applications in home, work place and in the external environment. Uses of electromagnetic energy increase globally and involve valid public health concerns about possible related health risks. The power output of mobile phones is a matter of particular concern. This concern encourages study of the effect of EMF on biological cells and more attempts have been undertaken to understand the underlying mechanisms. When biological cells are exposed to EMF, their compartments and the adjacent media absorb energy. The energy absorption, *at a specified frequency or wavelength*, is known as the Specific Absorption Rate (SAR) which is defined as the ratio of the absorbed power per unit mass of tissue, expressed in W kg^-1^. The National Council on Radiation Protection and Measurements (NCRP) defines SAR as the incremental power absorbed by an incremental mass of the tissue contained in a volume element of a given density. It is dependent on the internal electric field (EF_i_) generated within the tissue and the specific conductivity of the tissue, σ (S m^-1^) [1]. In the human body, since every tissue or group of organs consists of many different types of biological material the SAR varies significantly between different biological tissues and even within a single type of tissue [2, 3]. Conductivity varies for different tissues and different SAR levels have been reported. Most studies have tried to report SAR for whole body, head and bone [2, 3] but these are all very heterogeneous experimental material. However, there is very little work has done on SAR calculation for human red blood cells (HRBC), even though the HRBC cell is an important functional body fluid and is readily accessible for experimentation. Blood delivers oxygen to the vital parts and transport nutrients, vitamins, and metabolites. It also is a fundamental part of the immune system. Its dielectric parameters are of relevance for various medical applications, like cell separation (e.g. cancer cells from normal blood cells [4, 5], checking the deterioration of preserved blood in blood banks [6], and dielectric coagulometry [7]. Precise knowledge of the dielectric properties of blood is prerequisite for fixing limiting values for electromagnetic pollution (via the conductivity parameters intrinsic to SAR calculations) [8, 9]. Previous work done by Simeonova et al. [10] investigated cellular energy absorption of HRBC influenced by molecular properties, assuming a cytoplasmic conductivity (σ_c_) of about 0.53 S m^-1^. They found energy absorption in HRBC cytoplasm higher than model calculations at frequencies above 500 MHz. They pointed out that the standard conductivity they used in their model may change dependent upon on cell water and internal ionic concentrations. In addition, it has been known that a small change in the surrounding environment such as temperature or external ionic concentration will disturb HRBC cytoplasmic conductivity, especially cell shape and cell volume [11, 12]. Our previous paper [13], showed that increase in temperature leading to cell water cell volume and cytoplasmic conductivity changes generated higher energy absorption in HRBC. Volume change influenced by external ionic concentrations of external media suspension (blood plasma) was reported by Malka et al. [14]. Any volume change of the cells leads to an alteration of the concentration of all intracellular solutes, even if no net flux of them into or out of a cell has occurred [15, 16]. HRBC cytoplasm is highly complex: it contains large amounts of salts, haemoglobin, proteins, nucleic acids, and smaller molecules which can easily be changed by external conditions [15, 17, 18]. Change in external medium leads to cell volume change to adjust osmotic pressure and consequently disturbs cytoplasmic conductivity. To remain osmotic pressure, ionic concentration and other solutes between the cell and its environment need to balance. Aquaporins in the HRBC membrane facilitate very rapid osmotic adjustment and stabilisation of cytoplasmic conductivity (σ_c_) [19] and hence rapid volume and shape adjustment in HRBC. Na^+^ cation is the major ion in blood plasma and a major cation of the HRBC cytoplasm and plays an important role in cytoplasmic conductivity and osmotic pressure.

Several methods have been proposed to obtain HRBCs dielectric parameters. Most research has addressed only limited aspects, including electrical impedance [20], erythrocyte suspension in an electric field [21], and haematocrit volume dependency [22]. Furthermore, most of the studies have been performed using blood containing anticoagulant agents from blood banks [23] and single cell dielectric spectroscopy such as electrorotation [24, 25]. However, there are still many interpretive problems from such experiments remaining to be solved. For example, the often unsuspected effect of the Joule heating occurring during the investigation consequently disturbs cell volume, cytoplasmic conductivity, osmotic pressure, and ionic decreases in the cytoplasm. To avoid such problems, modelling cells with constant volume and constantly held temperature are needed [24–26]. However, the available data has limited scope and there are still unclear aspects of the dielectric properties of blood cells. This paper aims to investigate the effects of [Na^+^] influence cytoplasmic conductivity which is used for SAR calculation in HRBC.

Cytoplasmic conductance is a difficult-to-access parameter in most types of cells, usually only experimentally measurable in giant cells [27] and yet is an important measurement to have in dielectric studies of cells. In our previous study we have shown that estimates of cytoplasmic conductance in HRBC can be made based on very simple experimental measurements [13]. In the present study, we show the effects of blood plasma Na^+^ concentration and plasma osmolality at standard benchmark (25°C) and physiological temperatures (37°C) and their effect upon electromagnetic radiation absorption (SAR) by HRBC. Current guidelines for permissible SAR do not take human and mammalian body temperature into proper account.

## 2. Theoretical approach

Our theoretical approach is based upon our previous paper [13]. The full development of the models used in the present study are outlined in a pdf as Supplementary Material. This theoretical approach could not be applied to a more complex medium such as physiological saline or blood plasma and so the simple NaCl system containing only two major ions was used. Na^+^ and Cl^-^ are major ions in the blood plasma and are important ions in the HRBC cytoplasm.

## 3. Materials and methods

### 3.1 Human erythrocytes

Five separate lots of whole blood from healthy donors was collected from the blood bank (Suratthani Hospital, Thailand), stored no longer than 3 days at 4°C were investigated. No preservatives were used. To obtain erythrocytes, the cells need to be separated from blood plasma by centrifugation (H1850, Cence, Hunan, China) at 2000 *g* for 8 min. The supernatant plasma was removed with a syringe and the sediment (erythrocytes) was kept for the cell water and volume experiments.

### 3.2 Preparation of suspending solutions

Isosmotic solutions with osmolar activity (γ) of 308 mOsmol kg^-1^ at osmotic concentration (c) of NaCl and sucrose solutions both containing of 1 mM sodium phosphate buffer (pH 7.4) were prepared. Their ionic concentrations dissolved in the solutions were calculated from their osmolar activity (γ) and osmotic coefficient (φ) in the relation γ = φ×c [28], where φ of NaCl and sucrose solutions were 0.93 and 1, respectively. Isotonic NaCl is hence 148 × 2 × 0.93 = 275 mOsmol kg^-1^ and isotonic sucrose is 275 mM Sucrose. The calculated concentrations of sodium and chloride were 148 mM, the NaCl solution has a conductivity of about 1.43 S m^-1^. Four experimental NaCl solutions were selected for the following reasons (1) 0.53 S m^-1^ as it is the HRBC cytoplasmic conductivity (made isotonic by added sucrose), (2) 1.00 S m^-1^ as it is the conductivity of blood plasma under hyponatremia [29], (3) 1.48 S m^-1^ as it is the normal physiological conductivity of blood plasma and (4) 2.00 S m^-1^ as it is conductivity of blood plasma under hypernatremia. Isotonic NaCl and sucrose solutions were mixed to adjust the conductivities of lower than 1.43 S m^-1^. The concentrations of NaCl experimentally obtained are presented in the Table 1 together with their ionic strength. All solutions were prepared at the temperature compensation mode (20°C) of the conductometry meter used (MC 126, Mettler-Toledo, Greifensee, Switzerland). Experimental temperatures were carried out at 25°C (routine benchmark temperature for experimental data) and 37°C (physiological body temperature).

**Table 1:** Ionic concentrations and ionic strengths of NaCl solutions for four measured solutions measured at 25°C.

σ_s (S m_^-1^_)_	[NaCl] (mol L^-1^)	Ionic Strength (mol L^-1^)
0.53	0.0584 (Isotonic by added isotonic sucrose solution)	0.0584
1.00	0.1154 (Isotonic by added isotonic sucrose solution)	0.1154
**1.43**	**0.1483**	**0.1483**
1.70	0.1700	0.1700
2.00	0.2023	0.2028

The Ionic Strength was calculated according to Supplementary Eq. (3) in S2 File. Low NaCl solutions that were isotonic were prepared by adding isotonic sucrose to maintain isotonicity (275 mOsmol kg^-1^). Standard conditions in **bold**.

### 3.3 Running experiments and collecting cells

The amount of experimental solutions was calculated that was needed for the dilution of 50 μl of the erythrocytes to a final cell concentration of 10% (v/v). The cells were then incubated in the experimental suspending solution for 5 min and the *Hct* of the suspensions were determined by haematocrit-centrifugation at 10,000 *g* for 8 min. The short incubation time allowed enough time for osmotic adjustment via water flow through aquaporins [19] but not enough time for substantial net ion fluxes. In parallel, the cell number was microscopically determined using a standard haemocytometer (Improved Neubauer, Marien Field, Lauda-Koenigshofen, Germany). After the haematocrit-centrifugation and cell number was obtained, mean cell volume (MCV) was determined following standard clinical methods [30, 31]. No significant lysis of the cells was observed because the supernatant of the centrifuged cells in the haematocrit tube was clear. Some plasma is trapped between red cells after the blood has been centrifuged, however the International Council for Standardization in Haematology ICSH [32] has recommended that in routine practice no correction should be applied.

### 3.4 Cell volume determination

After the cells were spun down at 10000 × g for 8 min, the sedimentation of erythrocytes was taken into a standard 1.5 ml Eppendorf tube filled with experimental solutions and suspended for only 5 min. The suspension was spun down at 10,000 *g* for 8 min. A 300 μl of the packed cell volume was then pipetted into a pre-weighed test tube [30, 31]. In parallel, the haematocrit cell volume (*H*_*ct*_) was determined experimentally. The tube was weighed before and after drying at 80°C for 24 h. The cell water (*W*_*c*_) was calculated from the total wetted cells mass (*W*_*t*_) and dried cells mass (*W*_*d*_) [31] as:

$$\%\;W_{c}=\frac{W_{t}-W_{d}}{W_{t}}\times 100-\left(\frac{100-Hct}{Hct}\right)\times 100$$ (1)

### 3.5 Cytoplasmic conductivity and conductance calculation

Cytoplasmic conductivity of human erythrocytes were calculated according to Pauly and Schwan [17] using known concentrations of different ionic species and limiting ionic conductance values at 25°C presented in Bockris et al. [33]. The internal viscosity (F068) of 5.91 mPa s at 37°C [34] was introduced into Walden’s law (see S2 File). The calculated cytoplasmic conductivities are presented in the fifth column of the Table 2. The effective conductivities in column six were obtained from the difference between conductivity at finite dilution (column four) and of cytoplasmic conductivity influenced by viscosity (column five). Under routine conditions the volume effect of haemoglobin is not considered because it can be neglected. However, when the erythrocytes are suspended in external media with conductivities different from that under physiological conditions, the cell volume change has to be considered. We used NaCl as a simple approximation for blood plasma.

**Table 2:** Calculation of the internal conductivity of HRBC under physiological conditions using known concentrations of different ionic species and limiting ionic conductance values at 25°C by Bockris et al. [33].

	(1)	(2)	(3)	(4)	(5)	(6)
Ion						
Cl^-^	0.055	75.88	0.42	47.85	0.26	0.15
HCO_3_^-^	0.015	44.27	0.07	27.92	0.04	0.02
X^-^	0.01	35.61	0.04	22.46	0.02	0.01
HbO_2_^-^	0.042	26.63	0.11	16.79	0.07	0.04
Na^+^	0.0186	49.86	0.09	31.44	0.06	0.03
K^+^	0.095	72.97	0.69	46.02	0.44	0.26
½ Mg^++^	0.0051	52.97	0.03	33.41	0.02	0.01
Total	0.2407	358.19	1.44	225.90	0.91	0.53

The internal viscosity of 5.91 mPa s at 37°C was introduced into Walden’s law [34].

Key:

(1) Intracellular Concentration (mol l^-1^)

(2) Limiting Ionic Conductance (x 10^−4^ S m^2^ mol^-1^)

(3) Conductivity Contribution (S m^-1^)

(4) Limiting Ionic conductance influenced by Viscosity (x 10^−4^ S m^2^ mol^-1^)

(5) Effective Conductivity Influenced by Viscosity (S m^-1^)

(6) Effective Conductivity Influenced by Cell Water (S m^-1^)

### 3.6 Statistics

Zar [35] was used as the standard statistical text. ANOVA-based Tukey tests (p < 0.05) for multiple comparisons. The data set is included as an attached PDF file.

## 4. Results

### 4.1 Influence of suspending solutions on cell volume and cell water

**Fig 1:**
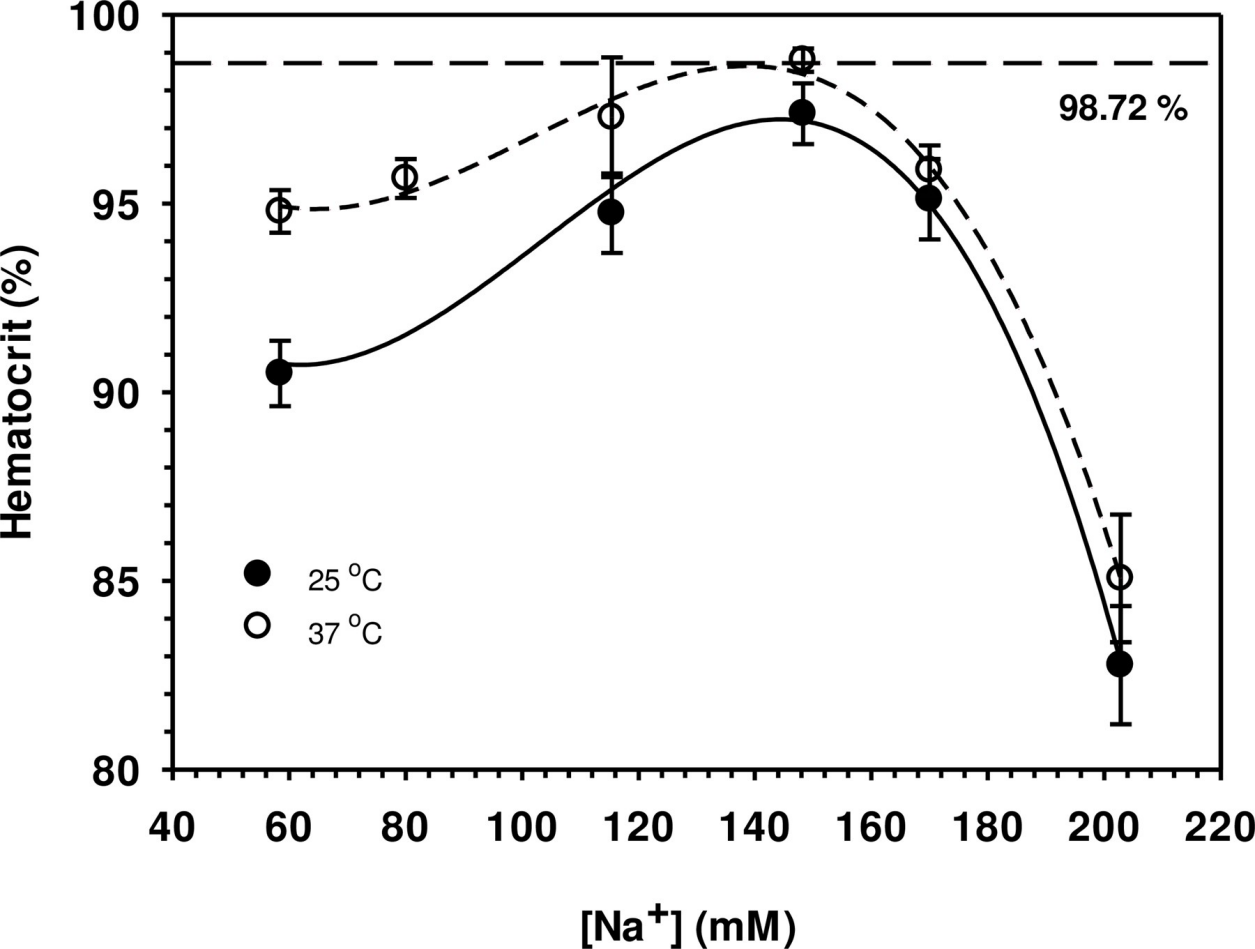
Haematocrit of red cells suspended in 5 different [NaCl] at 25 and 37°C compared to that in the physiological state in which [NaCl] is 148 mM and temperature 37°C (the reference lines on the graphs). The haematocrit volume plasma was 100% and 98.72% in 148 mM NaCl at 37°C. Error bars are ±SE, means based on five replicates (5 separate individuals): the fitted lines are spline curves [35]. The Tukey test interval for testing for significant (p < 0.05) differences between any two mean values was V% = 5.513: V% at each [Na^+^] at 25 *vs*. 37°C were not significantly different under control and higher [Na^+^] but diverged at 25°C (lower) and 37°C (higher) under the lowest (58 mM Na^+^).

**Fig 2:**
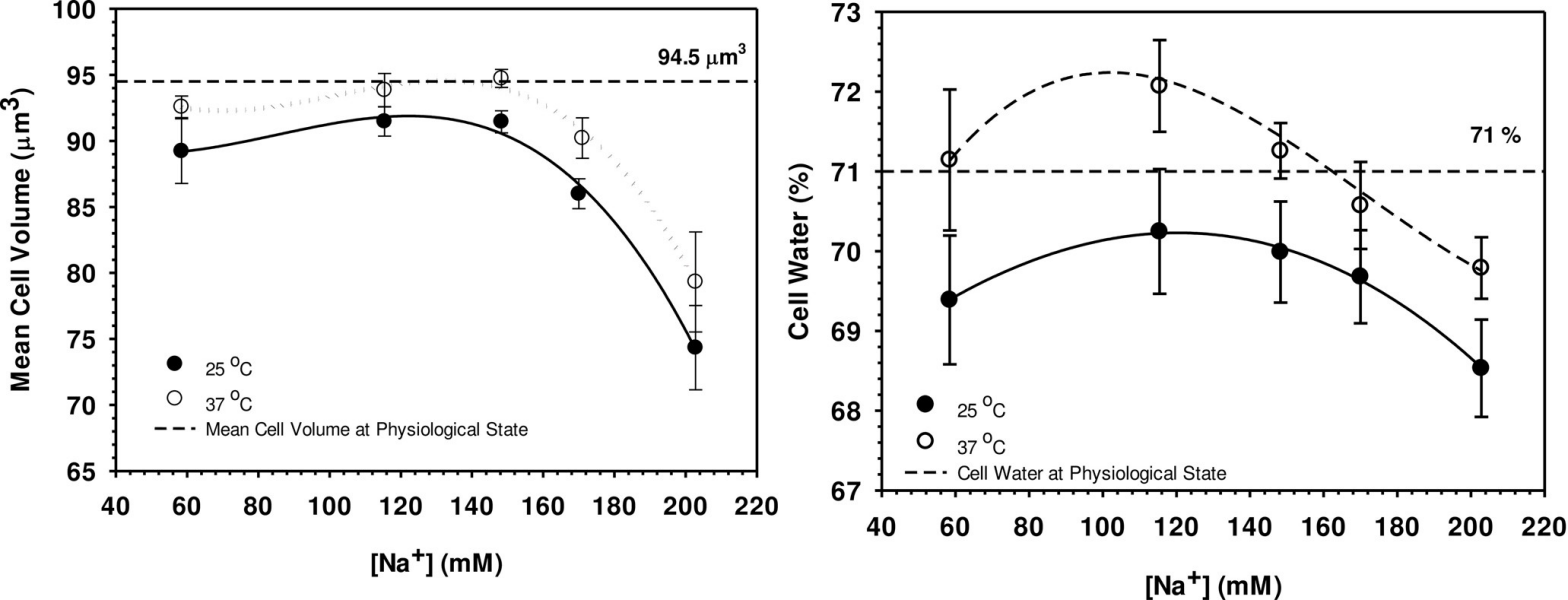
Measured mean cell volume (A) and relative cell water content (B) of HRBC cells suspended in 5 different [NaCl] at 25 and 37°C compared to that of the physiological cell volume value of 94.5 μm^3^ (the reference lines on the graphs). Error bars are ±SE, means based on 5 replicates (5 separate individuals): fitted lines are spine curves [35]. The Tukey test interval for testing for significant (p < 0.05) differences between any two mean cell volume values (Fig 2A) was V_c_ = 9.50 μm^3^: V_c_ at the two temperature were not significantly different but at [Na^+^] above the control concentration (148 mM) there was significant decreases in cell volume. The Tukey test critical value for cell water (Fig 2B) was 3.04%. Generally cell water was higher at 37°C but was only significantly higher at lower [Na^+^] (58 and 115 mM).

**Fig 3:**
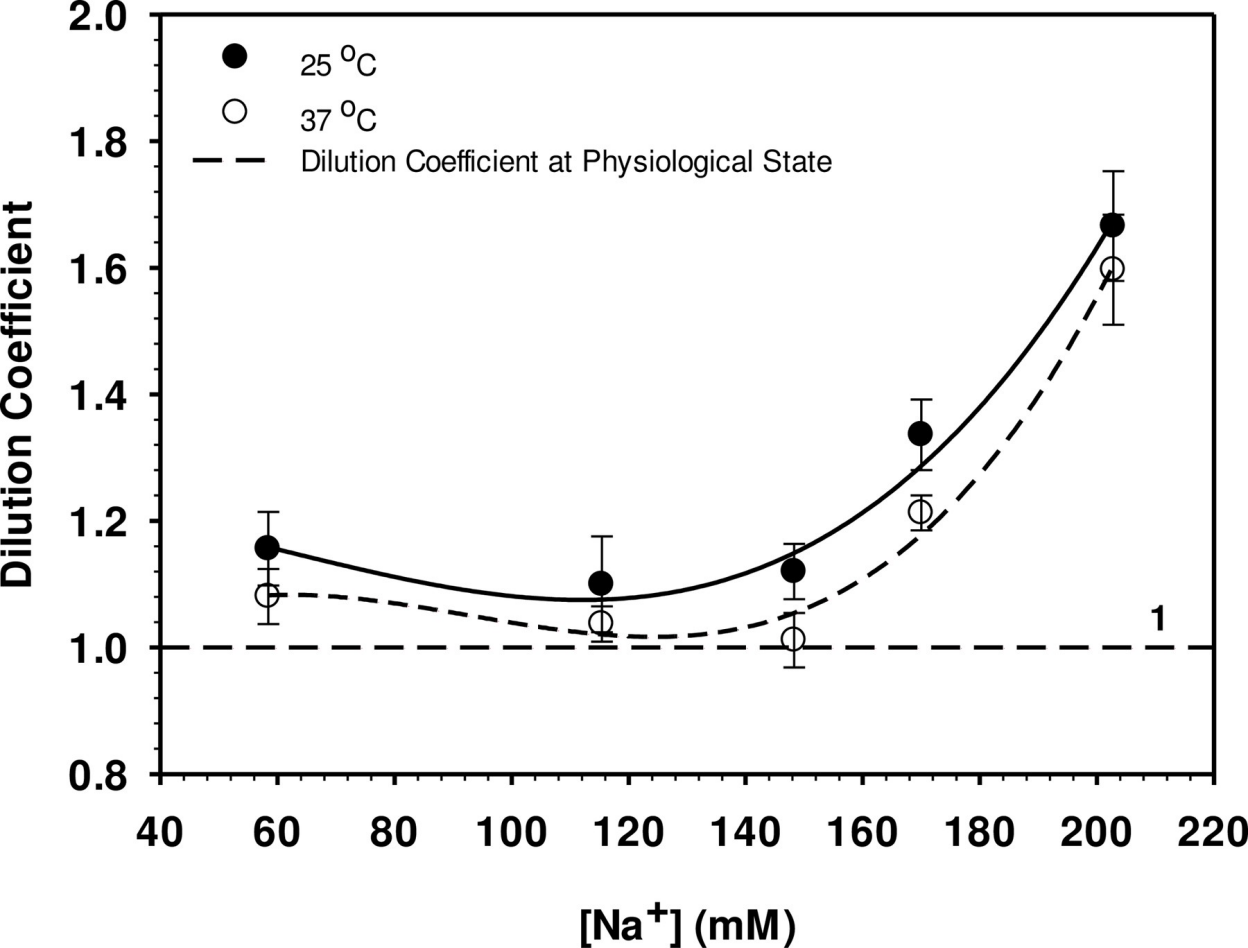
Dilution coefficient (τ) at 25°C and 37°C for 5 suspending media compared to that of a physiological value of τ = 1. Error bars are ±SE, means based on five replicates (5 separate individuals): the fitted lines are spline curves [35]. The Tukey test interval for testing for significant (p < 0.05) differences between any two mean dilution coefficient values was **τ** = 0.278: **τ** at the two temperatures were not significantly different but at [Na^+^] above the control concentration (148 mM) there were significant increases in **τ** at both temperatures.

Using the haematocrit-capillary method together with determination of the cell number as described in Materials and Methods, the experimentally determined haematocrit values obtained are shown in Fig 1. 100% is the haematocrit volume found in human plasma and 98.72% was the volume found in 148 mM buffered NaCl. ANOVA-based Tukey test procedures [35] have been used to identify significant differences (p < 0.05) between mean values in all of the figures. The fitted lines on all the figures are spline curves [35]. The cell volume of about 94.5 μm^3^ (Fig 2A) with a relative water content of 0.71 (Fig 2B) were found at physiological conditions (red blood cells with blood plasma conductivity around 1.48 S m^-1^). These findings agree very well with those previously published [36–38]. When erythrocytes were suspended in experimental solutions, changes in cell volume and cell water vary according to the conductivity of the external solution, or in other words concentrations, of NaCl or NaCl + sucrose. In hypotonic and isotonic solutions with conductivities of 0.50, 1.00 and 1.48 S m^-1^ ([NaCl] of 58.3, 115.4 and 148.3 mM, respectively), the cell volume is almost constant at temperatures 37°C but it is smaller at 25°C. In hypertonic solution (2.00 S m^-1^ in 223.8 mM NaCl), however, smaller cell volumes were observed at both temperatures. As can be seen from the results, the process of volume adaptation is reflected by changes in the cell water content (Fig 2B). Shift of both parameters from the physiological state provide the dilution coefficient (τ) as given by Supplementary Eq. 5 in S2 File and is shown in Fig 3. In hypotonic and isotonic solutions (0.50–1.48 S m^-1^), the coefficient stays almost constant and is increased at conductivities of 2.00 S m^-1^ under which cell water and cell volume are both reduced.

### 4.2 Influence of suspending solutions on cell shape

**Fig 4:**
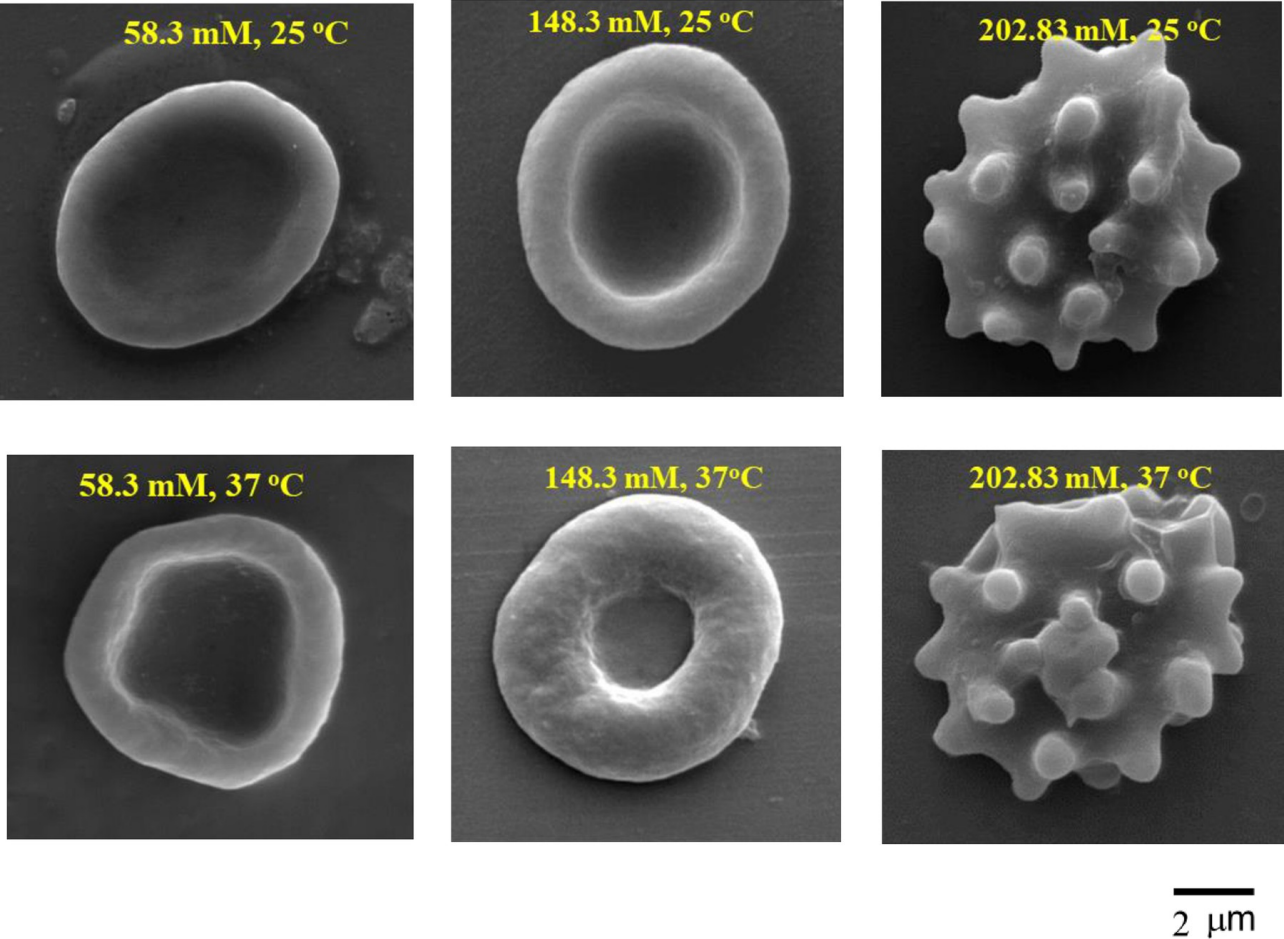
Electron micrographs of HRBCs shapes in 3 selected suspending solutions of isotonic solutions containing [Na^+^] of 58.4 and 148.3 mM and hypertonic solution (202.83 mM). Normal erythrocytes left and centre, echinocytes on right. Normal erythrocytes closely approximate a Cassini disk.

The shape of HRBCs can be drastically changed by the external media [39, 40] even using the very short incubation times used in the present study which allowed time for water to reach new equilibria but precluded substantial ion transport. Selected scanning electron micrographs of cells suspended in a range of experimental solutions at temperatures of 25°C and 37°C are shown in Fig 4. For isotonic solutions, with conductivities of 0.53 S m^-1^ and 1.483 S m^-1^ at a temperature of 25°C, the cells maintained the appearance of normal erythrocytes (discocytes). In hypotonic solution they were still discocyte in shape although different in appearance. When external solutions are changed to hypertonic conditions (2.02 S m^-1^) echinocytes (Fig 4) were observed at both temperatures. As erythrocytes undergo cell volume changes while the surface area of the membrane remains constant [39, 40] any change of cell shape disturbs the cell volume [41–43]. Echinocyte formation is reversible if the cells are subsequently resuspended in isotonic solution with no apparent permanent damage.

### 4.3 Influence of suspending solution on cytoplasmic conductivity

Cytoplasmic conductivity of human erythrocyte was calculated according to Pauly and Schwan [17]. The internal viscosity of 5.91 mPa s at 37°C [34] was introduced into Walden’s law. The calculated cytoplasmic conductivities are presented in the fifth column of the Table 2 [13]. The calculated cytoplasmic conductivity was 0.53 S m^-1^. This value agrees very well with those reported first in Gimsa [42], then by Simeonova et al. [10] and later by Panagopoulos et al. [2]. The same calculation procedure carried out in our previous paper [13] was then applied to the range of conditions described in the present paper.

**Fig 5:**
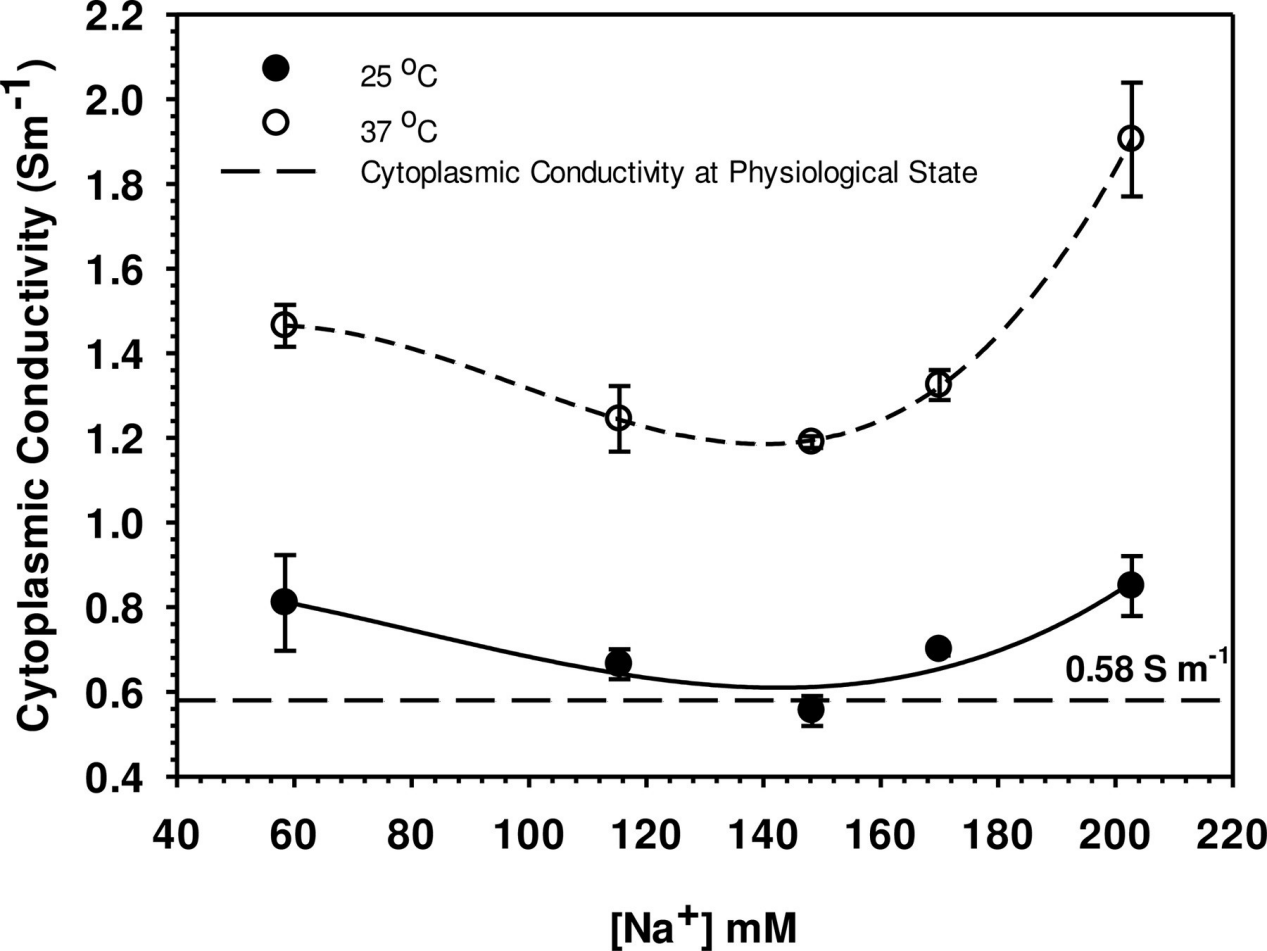
Calculated cytoplasmic conductivities over [Na^+^] of suspending solutions at two desired temperatures. The calculations were carried out as that presenting in Table 2. The volume effect of haemoglobin has been allowed for by the introduction of the dilution coefficient term (τ) in Supplementary Eq. (5) in S2 File into ionic concentrations presented in column 2 of Table 2. The conductivity of normal blood has a conductivity of about 1.48 S m^-1^. Values are means ± SE (n = 5, 5 separate individuals): the fitted lines are spline curves [35]. The Tukey test interval for testing for significant (p < 0.05) differences between any two mean cytoplasmic conductivity values was 0.340 S m^-1^: conductivity was higher at 37°C than at 25°C at all [Na^+^] tested. Conductivities at the two temperatures were not significantly different but at [Na^+^] above the control concentration (148 mM) there were significant increases in conductivity at both temperatures.

Cytoplasmic conductivity was calculated as follows: First a constant haemoglobin volume per cell of V_Hb_ = 28.4 μm^3^ was calculated, assuming a cell volume of 98 μm^3^ [37] or a volume share of about 29%. This volume is assumed to be “electrically inert”. For the physiological state of cytoplasmic conductivity (1.48 S m^-1^), 100% total volume of V_std_ = 94.5 μm^3^ as the standard state, a cell water volume of V_w_ = V_std_-V_Hb_ = (94.5–28.4) μm^3^ = 66.1 μm^3^. It was assumed that changes in the overall cell volume are solely due to changes in V_w_ at a constant external ion concentration (V_Hb_ is constant) and c_std_ is the concentration of ions in the cell water under standard conditions. The physiological ion concentration in the cytoplasm under standard conditions would be c_std_×V_w_/V_std_ = c_std_ because V_w_/V_std_ is unity. For a different cell volume (V_c_), the actual concentration in the cytoplasm becomes c = c_std_×V_w_/V_std_ where V_w_/V_std_ is not unity. Neglecting the conductivity contributions of the osmolytes present in small quantities, the cytoplasmic conductivity at any volume change was calculated by multiplying the cytoplasmic concentration of all ionic species corrected by the dilution coefficient (τ) with their molar conductivities (Λ_i_; Supplementary Eq. 1 in S2 File). Calculated cytoplasmic conductivities over a range of external media conductivities are shown in Fig 5.

### 4.4 Calculated energy absorption

**Fig 6:**
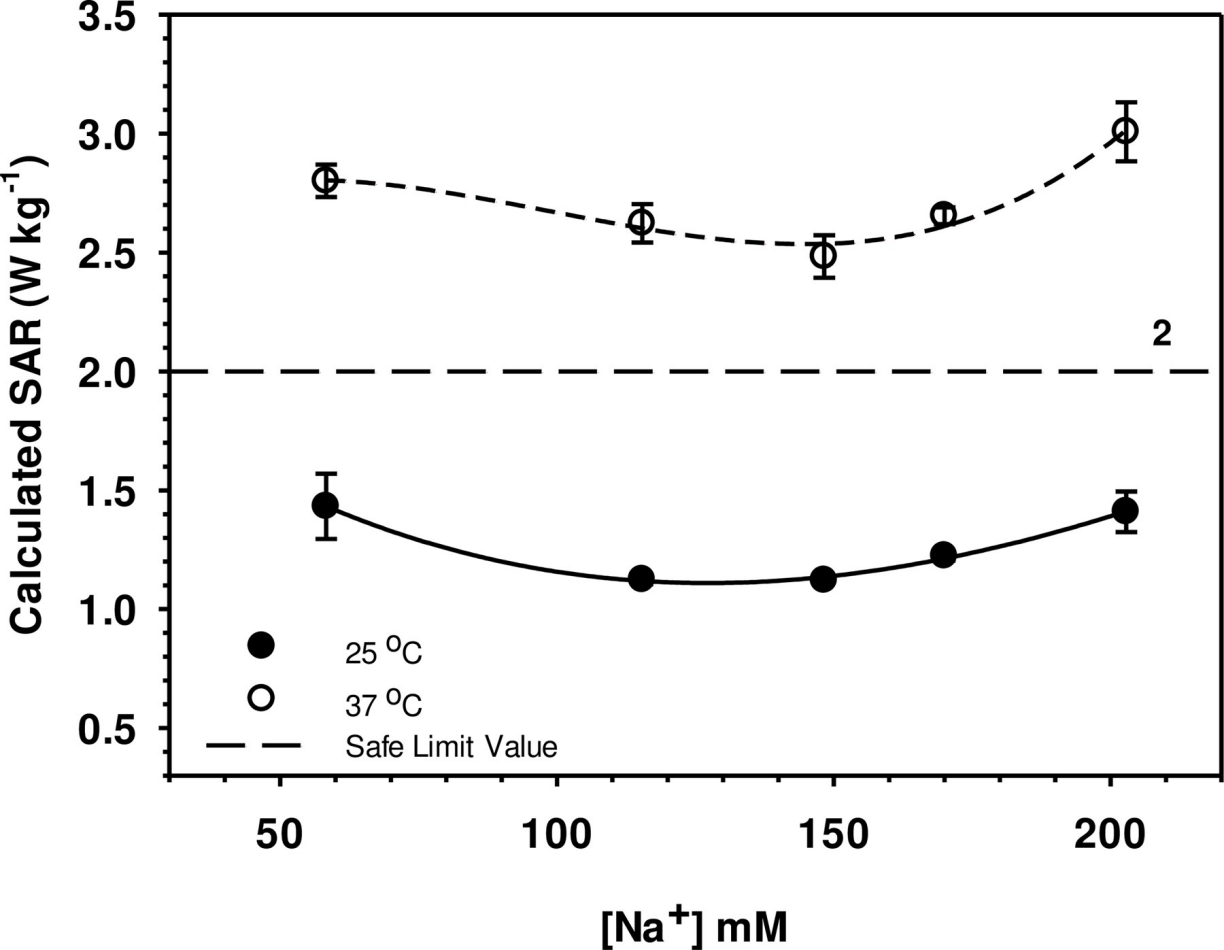
Calculated SAR of cytoplasm of HRBC over [Na^+^] at standard laboratory benchmark temperature and normal body temperature. The prescribed upper *legally* safe limit guideline is 2.0 W kg^-1^ for 10 g body tissue for 900 MHz EM (dotted line). Error bars are ±SE, means based on 5 replicates (5 separate individuals): the fitted lines are spline curves [35]. The Tukey test interval for testing for significant (p < 0.05) differences between any two mean values was SAR = 0.399: SAR at each [Na^+^] were all different at 25 *vs*. 37°C but SAR at 25°C and 37°C were generally not significantly different with the exception of SAR at 37°C and at control (148 mM) Na^+^ and at 202 mM Na^+^.

SAR values of erythrocytes suspended in experimental solutions with different [NaCl] were calculated from Supplementary Eq. (8) in S2 File normalized to an external field of 1 Volt m^-1^ are presented in Fig 6.

## 5. Discussion

### 5.1 The role of sodium ions

The human body requires Na^+^ to function properly. It takes on several functions once ingested and dispersed into the blood stream and other fluids of the body. Na^+^ regulates the electrolytes allowing the brain cells to carry out electrical signalling and control the body through the nerves and muscles. It also controls fluid levels, such as the total blood volume, which subsequently affects blood pressure. In the normal case, [Na^+^] in blood plasma is about 123 to 140 mM [44]. Deviation from these values are hyponatremia (with the [Na^+^] of less than 120 mM [29] or hypernatremia ([Na^+^] higher than 160 mM). Both hyponatremia and hypernatremia can lead to the possibility of abnormal blood function. In particular, hyponatremia, defined as low [Na^+^] in blood is caused by a disorder of water balance, with a relative excess of body water compared to the total body sodium. Whereas, hypernatremia results in the appearance of edema resulting ultimately in kidney and heart failure. Recently it has been reported that the low blood sodium may significantly increase the risk and severity of coronavirus (SARS-CoV-2) infection [45]. In addition, the deviation of [Na^+^] in blood plasma disturbs osmotic pressure between the blood cells and blood plasma leading to a change in their physiological properties such as cell water and cell volume. Alteration of either or both parameters changes the cytoplasmic conductivity. The conductivity of HRBC is very important for electromagnetic medical applications such as imaging, hyperthermia and ablation [46]. The cytoplasmic conductivity of HRBC arises from various dynamic processes. Moreover, reliable measurement and determination of SAR in the human body is not possible without precise knowledge of the conductivity of the tissue in question [47]. In general, red blood cells are normally enclosed in a high resistance membrane acting as shielding from external electric power distribution into the cytoplasm. However, if the cells are exposed in a high frequency external electric field, the membrane conductivity breaks down (called in electrophysiological jargon “punch through”). This changes the energy absorption rate and may disturb SAR level in the red blood cell if they are being exposed to medical electromagnetic equipment such as X-rays [48]. High electromagnetic field intensity emitted from X-ray equipment may increase cytoplasmic conductivity and consequently SAR of HRBC in case of hyponatremia or hypernatremia or even in normal blood. Increase of SAR values of blood at 37°C ranging from 0.173–1.417 W kg^−1^ by irradiation with 532 nm laser power of 50 mW for 5–10 min has been reported [49].

### 5.2 Influence of suspending solutions on cell volume and cell water

When erythrocytes are suspended in media which differ from that of the physiological state (1.48 S m^-1^), rapid cell volume changes were observed (Fig 2A). Lightly buffered NaCl [13] was deliberately chosen as the suspending electrolyte for the present study because Na^+^ and Cl^-^ are the dominant ions present blood plasma and so NaCl was the simplest system in which to study cell volume changes in HRBC and is the electrolyte of choice for such studies [4, 17, 21, 22, 40, 41, 50, 51] although the role of K^+^ should not be overlooked [52, 53]. The time course of experiments in the present study were very short (5 minutes) which would not have allowed much time for substantial leakage of K^+^ or other ions. In hypertonic solutions, decreases in cell volume and cell water were observed with rising external ionic strengths. Hypotonic solutions did not simply increase cell volume over the experimental range used, contrary to what one would naturally expect. The HRBC is capable of regulating its volume (and importantly also cell shape) over quite a wide range of osmolality (Fig 2A and 2B). Hoffman [16] rightly points out that the HRBC membrane is not homogeneous: the rim is more rigid than the top and bottom of the cell (rather like a car tyre). Normal erythrocytes closely resemble a Cassini disk however the rigidity of the rim results in them being able to retain this shape over a wide range of osmotic potentials (Fig 4). Only in very hypotonic conditions does the HRBC cell simply swell up like a balloon and burst. Maximum volume and water content were found at a conductivity of 1.48 S m^-1^ (physiological value). The changes in cell volume for both cases depend mostly on water fluxes flowing through the cell membrane to balance the intracellular osmotic pressure, which is mostly altered by changes in the ionic concentration gradients across cell membrane. However, the mechanism of volume change in hypertonic and hypotonic solutions is different. In hypertonic solutions, cell shrinkage is caused only by water moving out of the [38, 54] accompanied by little or no net ion fluxes. For hypotonic conditions, no simple answer has been offered for regulation of cell volume. However, if the rates of ion transport (mainly K^+^/Na^+^ ATPase activity and balancing Cl^-^/HCO_3_^-^ exchange) are not correctly matched, cells will inappropriately shrink or swell which mostly results from a loss of osmotically active ions, usually K^+^ and Cl^-^ which is known to be very sensitive in cell volume change [38, 41, 43, 54]. Normally, exchange of Cl^-^ through the cell membrane is mostly to achieve charge balance and to balance the osmotic pressures across the membrane. In hypotonic solution, the parameter altering the balance of the system and drives Cl^-^ net fluxes across membrane is the pH and concentrations of ions in the external solution. pH of the external solution (pH_o_) however, influences charges on the haemoglobin molecules which are dependent upon the intracellular pH (pH_i_) [37, 55]. Under physiological conditions, haemoglobin is near its isoelectric point, pH_iso_ of 6.8 at 25°C. The total haemoglobin molecule is therefore uncharged at pH_iso_ (*Z*_*M*_ = 0). When the external pH is altered, the charges of haemoglobin become:

$$Z_{M}=-Z_{Mstd}(pH-pH_{iso})$$ (2)

where, Z_Mstd_ is buffer capacity of haemoglobin having a value of 10.5 eq/mol in the standard state. At intracellular pH lower than the isoelectric point (pH < pH_iso_), Z_M_ becomes positive, and vice versa for pH_i_ above it (pH > pH_iso_*)*. In our experimental case, the cells were suspended in media with a pH_o_ of 7.4 (physiological pH of blood plasma) [24], thus the net charge of the intracellular haemoglobin was slightly negative. To balance excessive negative charges and since Na^+^ and K^+^ are relatively less permeable [37], Cl^-^ would be expected to move of out of the cell decreasing intracellular [Cl^-^]. To balance osmotic pressure, water would then flow out the cell resulting in shrinkage. However, cell shrinkage caused by external pH alone cannot completely describe the overall of volume change. Other reasons should also be taken into the consideration. One should note that after Cl^-^ moves out from the cell to balance the excess negative charge from haemoglobin, the cell itself would balance both iso-osmolarity and electroneutrality according to the new Donnan equilibrium. *In vivo*, HRBCs have cationic charge concentrations of about +160 mmoles of charges/litre cell water. Diffusible anions (mostly Cl^-^) and phosphate compounds provide mobile negative charges of about -108 and -28 mmoles of charges/litre cell water respectively [37]. Haemoglobin has a molar concentration of 7 mmol/litre cell water with a charge concentration of -3.42 mol of charge/mol providing charge concentrations of about -24 mmoles of charges/litre cell water under physiological pH conditions [56]. Suspending the cells in external medium with pH 7.4, the charges of haemoglobin become negative with a molecule concentration of -8.4 charges/molecule (calculated from Supplementary Eq. 6 in S2 File) giving charge concentration of -28.72 mmoles of charges/litre cell water. These negative net charges result in the cell being out of neutrality with an excess charge concentration of -4.72 mmol of charges/litre cell water (Sum of +160–28.72–108–28). To neutralise these negative charges, Cl^-^ efflux occurs. At an external conductivity of 1.00 and 1.48 S m^-1^ however, the charge concentration of Cl^-^ outside is about 100 and 150 mmoles of negative charges/litre cell water (mmol e^-^ L^-1^), respectively which is higher than the interior positive charge +52.72 mmoles of charges/litre cell water). Cl^-^ therefore flows into the cell.

### 5.3 Influence of suspending solution on cytoplasmic conductivity

Few measurements or calculations of apparent cytoplasmic conductivity of HRBC are available for comparison with the present study [41]. Pauly and Schwan [17] attempted to calculate the internal conductivity of erythrocyte based on the idea that cytoplasm behaves like an electrolyte solution assuming the ions inside the cells experience neither relaxation (drag forces) nor electrophoretic effects. A calculated value of 1.44 S m^-1^ was obtained from the product of the ion concentrations in the cell and their molar conductivities (see column three in Table 2). The ionic balance in the human red blood cell from Pauly and Schwan [17] is given in the first columns of Table 2. The second column gives limiting ionic conductance for all ions of interest [57]. However, the *calculated* value is 2.7 times higher than the experimentally measured value of 0.518 S m^-1^ [17]. They hypothesized this discrepancy that was due to a decrease in the mobility of ions in the cytoplasm. Unfortunately, at that time, the cytoplasmic viscosity of HRBC had not been considered. Cokelet and Meiselman [34] concluded that the inside of a HRBC was better characterized as a non-aqueous solution because of the very high haemoglobin concentration and the volume it occupies in the cytoplasmic solution. Switching from considering the ions from being in a water environment to that of a non-aqueous solvent can alter their quantities by several orders of magnitude. The altered quantities including viscosity, dielectric constant of the electrolyte medium, the distance of closest approach of the solvated ions, and the radii of the solvated ions result in changes in the mobility of the ions at infinite dilution and the concentration of free ions which causes the conductance behaviour of an electrolyte to vary considerably.

To understand more about this phenomenon Walden’s Law (Supplementary Eq. 7 in S2 File) has been taken into consideration. A relative viscosity of cytoplasm of HRBCs with a value of 5.91 mPa s at 37°C [34] was applied. For comparison the viscosity of water at 37°C is 0.69 mPa s. Viscosity hinders the mobility of ions, and consequently reduces the cytoplasmic molar conductivities from those at infinite dilution (see column five of Table 2). The effective conductivities of cytoplasm corrected for viscosity are shown in column six of Table 2. The conductivities presented in column seven are the intracellular conductivities in comparison to conductivities at infinite dilution. The diminution of the conductivity may be result of the reduction in magnitude of the molar conductivity of ions (Λ_i_) according to an interionic interaction which reduces their conductivity to below that at infinite dilution (Λ^o^_i_). Including all the necessary correction factors, a cytoplasmic conductivity of 0.53 S m^-1^ could be calculated. This value agrees well with previous publications [17, 50, 51] and strongly confirms the predictions of Pauly and Schwan [17] that the discrepancy of internal conductivities obtained from the theoretical calculations and those obtained experimentally were due to the effects of viscosity. That is, the increased viscosity of the cytoplasm of the HRBC leads to a decrease in ion mobility at infinite dilution and hence *in vivo*. Recently, Hughes et al. [53] has documented that even though the membrane potential of HRBC is relatively small in magnitude (Δψ_i,o_ ≈-10 mV) it does have measureable effects on their surrounding blood plasma: thus it would be reasonable to assume that this electric field would have considerable effects within the much more restricted confines of the cytoplasm of the HBRC as well and affect cytoplasmic conductance.

Experimentally, as seen in Table 2, the cell volume can easily be changed by either a change in the external media or of temperature (Fig 2A). The changes in cell volume lead to alterations in cytoplasmic concentrations and consequently disturb the intracellular conductivity [24]. Cell swelling, caused by either the external media or temperature, result in a lowered conductivity. However, if the cell shrinks, higher intracellular conductivities will result. An adjustment of ionic concentrations in the cell to balance the osmotic pressure when they are suspended in a new environment seems to be the best description of this behaviour. Theoretically, an internal osmotic pressure is given by the sum of the number moles of the penetrable ions (n_ion_) and non-penetrable solutes (n_non-exch_*)* like haemoglobin. At equilibrium, the internal osmotic pressure is equal to that of the external medium [24]

$$\frac{n_{ion}}{V_{c}}+\frac{n_{Mol}}{V_{c}}=\text{constant}$$ (3)

If the cell volume (V_c_) decreases, the concentrations of the non-penetrant solutes (c_non-exch_) increases. However, Gary-Bobo and Solomon [56] showed that the increase in c_non-exch_ leads to a decrease in the net charge of haemoglobin. In this case, only the concentrations of mobile ions (c_ion_) play a role in balancing of the osmotic pressure. Thus, the increase in cytoplasmic conductivities of the shrunken cells can be attributed to the increase in c_ion_ to balance the net charges of haemoglobin. Inversely, if the cells swell, the concentrations of the c_non-exch_ decreases leading to a rise of the net charges of haemoglobin, hence to adjust the external osmolality, the concentration of mobile ions (c_ion_) falls, and generates a lowered conductivity. The cytoplasmic conductivity therefore mostly results from changes in the net charge of haemoglobin and penetrant solutes (K^+^, Na^+^, HCO_3_^-^ and Cl^-^).

### 5.4 Energy absorption and SAR safe limits

Specific Absorption Rate (SAR, W kg^-1^) needs to be calculated and quoted for specified EM frequencies and temperatures [3]. A typical mobile phone frequency is ≈900 MHz and the relevant temperature is 37°C *not* 25°C used in many lab-bench studies. Estimated SAR values for cytoplasmic erythrocytes suspended in external solution with varied [NaCl] are presented in Fig 6. In a suspending solution of [NaCl] 148.3 mM at 25°C and cytoplasmic conductivity of 0.58 Sm^-1^, the calculated SAR value is 1.25 W kg^-1^. The finding agrees very well with the work done by Rani et al. [3] who calculated SAR of human blood vary from 1.0 W kg^-1^ to 1.4 W kg^-1^ for external frequency of 900 MHz by assuming conductivity of blood of 1.58 Sm^-1^. Nevertheless, no data on SAR of cytoplasmic compartment of red blood cells have been reported when the cells are suspended in solutions and at temperatures of lower and higher than normal physiological conditions: these lead to upward shifts in cytoplasmic conductivities leading to higher SAR values. Previously we found SAR of human red blood cytoplasm to be 2.6 W kg^-1^ at the physiological temperature of 37°C in [NaCl] of 148.3 mM [13]: comparable to our results in the present study (Fig 6). This result is slightly above that which is acceptable by WHO [32] and International Commission on Non-Ionizing Radiation Protection, ICNIRP [58] guidelines that the SAR becomes harmful after 2.0 W kg^-1^ for 10 g body tissue. This is not surprising since the conductivity of HRBC cytoplasm at 37°C is not the same as the conductivity of whole blood (conductivity of 1.48 S m^-1^) but has a higher value. In suspending solutions of both higher and lower than physiological values, SAR increases to well above the prescribed safe limit (Fig 6). This finding suggests that the *legal* safe limit for SAR prescribed by the ICNIRP is *marginally safe* for normal healthy persons. However, when external conductivity increases, for example if the body has insufficient fluid intake and too much water loss occurs resulting in higher sodium concentration, hypernatremia of the blood plasma can occur. The SAR obtained from our calculations become considerably greater than the SAR safe limit and so is a matter of concern. In addition, it has been shown that when blood cells were exposed to higher frequency electromagnetic field, SAR becomes greater [3], perhaps because applied EM energy would be expected to affect membrane potential and hence intracellular conductance and the ionic balance of HRBC [53]. These should be taken into consideration when person whose blood is abnormal is treated by medicine equipment. The EM energy generated from equipment may harm their red blood cells.

## 6. Conclusions

This investigation found that the cytoplasm conductivity of HRBC is easily affected by cell volume changes. Cell volume of human erythrocytes is significantly affected by both external ionic concentrations and temperature. A proportional increase in cell volume depending on the external ionic strengths was observed in hypotonic solutions. In hypertonic solutions, the volume decreases were found. Changes in cell shape were found to depend on the composition of the external media more than temperature. In isotonic and hypotonic solutions, the cell remained a normally shaped erythrocyte. In hypertonic solution, echinocyte formation was observed. The cytoplasmic conductivity depends strongly on the external ionic strengths. This finding is not surprising physiologically but it has not been adequately taken into account in dielectric cell studies before.

Our study has shown that the cytoplasmic electrical conductivity of red blood cells can be estimated from very simple experimental observations *independently* of dielectric methods. This ability to estimate conductivity independently is important in validating dielectric experiments and for modelling the electrical behaviour of the HRBC. Cell volume of human erythrocytes is sensitively affected by both external ionic concentrations and temperature. The alteration of cell volume induces changes in the cytoplasmic conductivities which depend on external solution.

The results suggest that in future experiments on cellular mobility in strong electric fields special attention needs to be made to the composition of the external suspending solution, an increase in intracellular conductivity will be induced by osmotic pressure, especially at high media conductivities. The results of this study have clinical implications because some kinds of fevers cause changes in the ionic composition of the blood plasma which will affect HBRC conductivity and hence interfere with the ionic balance of HRBC.

A particular legal safe limit (at appropriate temperature) for SAR should be chosen such that maximum EM radiation that can sustain human health without introducing any biological changes. The SAR standards are regulated by world regulatory bodies like International Commission on Non-Ionizing Radiation [58].

## 7. Human ethics of investigation

Red blood cells of healthy donors were collected from the blood bank (Suratthani Hospital, Thailand) stored no longer than 3 days at 4°C were used experimentally. The blood was obtained from five volunteers at Suratthani Hospital in full accordance with medical ethics standards in Thailand. All blood was HIV tested before being supplied to us.

## Supporting information

S1 File (PDF)

S2 File (PDF)

## Abbreviations

C: osmotic concentration (Osmol kg^-1^)
c_ion_: concentration of ionic species (mM)
c_non-exch_: concentration of non-exchangeable solutes
c_std_: standard intracellular concentration of ions under standard conditions (mM)
EF_i_: internal electric field, {\displaystyle \mathbf {E} }within a tissue (Volts m^-1^)
Hct: haematocrit cell volume
MCV: mean cell volume
NCRP: National Council on Radiation Protection and Measurements
n_ion_: number of moles of potentially exchangeable ions
n_non-exch_: number of moles of concentrations non-penetrant solutes
pH_iso_: pH isoelectric point
SAR: Specific Absorption Rate (W kg^-1^)
V_w_: cell water volume
V_Hb_: volume inside HRBC occupied by haemoglobin
V_std_: total volume of cells in the standard state
W_c_: relative cell water content of cell
W_t_: wetted cell mass
W_d_: dried cell mass
Z_Mstd_: buffer capacity of haemoglobin in the standard state
σ: total conductivity (S m^-1^)
σ_c_: cytoplasmic conductivity (S m^-1^)
σ_s_: conductivity of a given solution (S m^-1^)
γ: osmolar activity
φ: osmotic coefficient
Λ_i_: equivalent molar conductivity of a specified ion (i) (S m^2^ mol^-1^)
Λ^o^i: equivalent molar conductivity of ion species (i) at infinite dilution (S m^2^ mol^-1^)
τ: dilution coefficient
η: viscosity
