# Supplementary material for: Influence of Na+ disorder on cytoplasmic conductivity and cellular electromagnetic (EM) energy absorption of human erythrocytes (PONE-D-21-36089)
Source: PLoS One. 2023 Feb 23;18(2):e0277044. doi: 10.1371/journal.pone.0277044 (PMC9949639; doi:10.1371/journal.pone.0277044)
Supplement: S1 File — (PDF) [file pone.0277044.s001.pdf]

PONE-D-21-36089
Influence of Na^+ Disorder on Cytoplasmic Conductivity and Cellular Electromagnetic (EM) Energy Absorption of Human Erythrocytes – SUDSIRI, CJ & RITCHIE, RJ. 2022

Data for Figure 1: Haematocrit of red cells suspended in 6 different [NaCl] at 25 and 37 °C

	[Na+] (mM)					
data (25 °C)	58.40	80.00	115.40	148.30	170.00	202.83
1	91.651	92.000	93.266	96.561	95.000	80.529
2	90.909	91.000	94.222	98.909	96.300	83.529
3	90.774	92.000	95.263	96.957	95.500	84.365
4	89.105	92.000	94.222	97.238	96.100	83.758
5	90.000	93.000	95.238	97.238	94.200	83.333
mean	90.488	92.000	94.442	97.381	95.420	83.103
SE	0.434	0.316	0.373	0.402	0.381	0.666
data (37 °C)						
1	95.592	96.000	98.108	99.238	95.600	83.000
2	94.561	95.000	99.266	98.333	96.400	86.923
3	94.774	96.000	95.434	98.954	95.200	86.947
4	94.000	96.000	96.263	98.909	96.800	85.235
5	95.263	96.000	96.000	98.774	95.200	83.333
mean	94.838	95.800	97.014	98.842	95.840	85.088
SE	0.277	0.200	0.720	0.148	0.325	0.845

Data for Figure 2A: Measured mean cell volume

	[Na^+] (mM)				
data (25 °C)	58.40	115.40	148.30	170.00	202.83
1	95.592	96.000	98.108	99.238	95.600
2	94.561	95.000	99.266	98.333	96.400
3	94.774	96.000	95.434	98.954	95.200
4	94.000	96.000	96.263	98.909	96.800
5	95.263	96.000	96.000	98.774	95.200
mean	94.838	95.800	97.014	98.842	95.840
SE	0.277	0.200	0.720	0.148	0.325
data (37 °C)					
1	95.592	98.108	99.238	95.600	83.000
2	94.561	99.266	98.333	96.400	86.923
3	94.774	95.434	98.954	95.200	86.947
4	94.000	96.263	98.909	96.800	85.235
5	95.263	96.000	98.774	95.200	83.333
mean	94.838	97.014	98.842	95.840	85.088
SE	0.277	0.720	0.148	0.325	0.845

Data for Figure 2B: Relative cell water content (B)

	[Na^+] (mM)				
data (25 °C)	58.40	115.40	148.30	170.00	202.83
1	69.560	70.655	69.952	70.300	68.157
2	68.405	70.877	70.775	69.500	68.109
3	70.623	69.302	69.017	69.100	69.234
4	69.200	69.500	70.000	69.200	68.000
5	69.157	70.903	70.203	70.300	69.157
mean	67.558	77.773	83.041	86.400	90.915
SE	0.361	0.350	0.284	0.262	0.273
data (37 °C)					
1	95.592	98.108	99.238	95.600	83.000
2	94.561	99.266	98.333	96.400	86.923
3	94.774	95.434	98.954	95.200	86.947
4	94.000	96.263	98.909	96.800	85.235
5	95.263	96.000	98.774	95.200	83.333
mean	94.838	97.014	98.842	95.840	85.088
SE	0.277	0.720	0.148	0.325	0.845

Data for Figure 3: Dilution coefficient at 25°C and 37 °C for 5 suspending media

	[Na^+] (mM)				
data (25 °C)	58.40	115.40	148.30	170.00	202.83
1	1.185	1.121	1.112	1.245	1.117
2	0.979	1.102	1.091	1.171	1.155
3	1.186	1.084	1.097	1.089	1.143
4	1.320	1.250	1.350	1.360	1.400
5	1.766	1.728	1.546	1.623	1.666
mean	1.287	1.257	1.239	1.298	1.296
SE	0.132	0.121	0.091	0.093	0.106
data (37 °C)					
1	1.106	1.008	0.993	1.230	1.662
2	1.016	1.075	1.075	1.200	1.681
3	1.105	1.029	0.980	1.180	1.515
4	1.096	1.037	0.998	1.240	1.530
5	1.104	1.023	0.995	1.241	1.523
mean	1.085	1.034	1.008	1.218	1.582
SE	0.017	0.011	0.017	0.012	0.037

Data for Figure 5: Calculated cytoplasmic conductivities over $[Na^+]$

	$[Na^+]$ (mM)				
data (25 °C)	58.40	115.40	148.30	170.00	202.83
1	0.890	0.690	0.580	0.690	0.800
2	0.730	0.640	0.530	0.710	0.900
3	0.820	0.610	0.510	0.730	0.920
4	0.800	0.690	0.570	0.750	1.010
5	0.860	0.680	0.530	0.690	0.980
mean	0.820	0.662	0.544	0.714	0.922
SE	0.027	0.016	0.013	0.012	0.036
data (37 °C)					
1	1.106	1.008	0.993	1.230	1.662
2	1.016	1.075	1.075	1.200	1.681
3	1.105	1.029	0.980	1.180	1.515
4	1.096	1.037	0.998	1.240	1.530
5	1.104	1.023	0.995	1.241	1.523
mean	1.085	1.034	1.008	1.218	1.582
SE	0.017	0.011	0.017	0.012	0.037

Data for Figure 6: Calculated SAR of cytoplasm of HRBC over $[Na^+]$

	[Na+] (mM)				
data (25 °C)	58.40	115.40	148.30	170.00	202.83
1	1.3357	1.1374	1.1137	1.237	1.4708
2	1.53	1.11	1.13	1.21	1.35
3	1.48	1.13	1.13	1.23	1.39
4	1.54	1.14	1.16	1.22	1.4
5	1.45	1.11	1.13	1.2	1.45
mean	1.467	1.125	1.133	1.219	1.412
SE	0.037	0.007	0.008	0.007	0.022
data (37 °C)					
1	2.7534	2.5661	2.5469	2.63	3.0956
2	2.85	2.68	2.42	2.68	2.92
3	2.81	2.59	2.43	2.68	3.11
4	2.78	2.6	2.55	2.59	3.02
5	3.01	2.61	2.54	2.6	2.98
mean	2.841	2.609	2.497	2.636	3.025
SE	0.045	0.019	0.030	0.019	0.036
