# Supplementary material for: Influence of Na+ disorder on cytoplasmic conductivity and cellular electromagnetic (EM) energy absorption of human erythrocytes (PONE-D-21-36089)
Source: PLoS One. 2023 Feb 23;18(2):e0277044. doi: 10.1371/journal.pone.0277044 (PMC9949639; doi:10.1371/journal.pone.0277044)
Supplement: S2 File — (PDF) [file pone.0277044.s002.pdf]

Theoretical Development: Influence of Na^+ Disorder on Cytoplasmic Conductivity and Cellular Electromagnetic (EM) Energy Absorption of Human Erythrocytes

Chadapust J. Sudsiri, Raymond J. Ritchie

Abbreviations:

$c_{non\text{-}exch}$ – concentration of non-exchangeable solutes,
c_{std} – standard intracellular concentration of ions under standard conditions (mM),
Hct – haematocrit cell volume,
MCV – mean cell volume,
pH_{iso} – pH isoelectric point,
SAR – Specific Absorption Rate (W kg^{-1}),
V_{std} – total volume of cells in the standard state,
W_c – relative cell water content of cell,
σ – total conductivity (S m^{-1})
σ_c – cytoplasmic conductivity (S m^{-1})
σ_s – conductivity of a given solution (S m^{-1})
Λ_i – equivalent molar conductivity of a specified ion (i) (S m^2 mol^{-1}),
$\Lambda^o{}_i$ – equivalent molar conductivity of ion species (i) at infinite dilution (S m^2 mol^{-1}),
τ – dilution coefficient,
η – viscosity.

Our theoretical approach is based upon our previous paper (Sudsiri and Ritchie, 2021). Total electrical conductivity of an electrolyte (σ, S m^{-1}) is the sum of individual conductivity of each ion in solution (σ_i, S m^{-1}) determined by its concentration of charges (c_i, mol l^{-1}) and equivalent conductivity (Λ_i, S m^2 mol^{-1}) (Pauly and Schwan, 1966) expressed as:

$$\sigma = \sum \sigma_i = \sum \Lambda_i c_i \qquad \text{(Suppl Eq. 1)}$$

The Λ_j decreases significantly with increase in the ionic concentration of all ions present. This relation is known as the Debye-Hückel-Onsager equation (Lide, 2002) and for an ideal system, Λ is given by:

$$\Lambda_i = \Lambda_i^o - (P + Q\Lambda_i^o)\sqrt{[I]} \qquad \text{(Suppl Eq. 2)}$$

where, Λ^o_i is the equivalent conductivity of ion (i) at infinite dilution (units of Sm^2 mol^{-1}). The parameters P and Q are constant parameters with values of 60.20 and 0.229, respectively (Lide, 2002) and [I] is the total ionic strength determined as:

$$I = \frac{1}{2}\sum_i c_i z_i^2 \qquad \text{(Suppl Eq. 3)}$$

where c_i is the molar concentration of the ions of type i which have the valence of z_i. For a uni-valent electrolyte such as NaCl (z = 1), the ionic strength is equal to the molar concentration.

The Λ_i^o can be the sum of the limiting ionic conductance, λ^0_{+i} and λ^0_{-j} (in S m^2 mol^{-1}) of positive and negative charges, respectively or can be forming by the product of ionic mobility (u_i) and Faraday's constant (F = 96485 C mol^{-1}) known as the Law of Independent Migration of ions given in the Eq (Suppl Eq. 4).

$$\Lambda_i^o = \lambda^0_{+i} + \lambda^0_{-j} = F(u_{+i} + u_{-j}) \qquad \text{(Suppl Eq. 4)}$$

Therefore, the electrical conductivity of the cell cytoplasm should give information about the state of the ions in the cell including free moving charges or bound ion such as proteins. Pauly and Schwan (1966) determined internal conductivity of erythrocytes and found a value of 0.518 S m^{-1} at the standard benchmark temperature (25 ^{o}C). *Note that this temperature is not the normal physiological temperature for the HRBC.* They concluded that the cytoplasmic conductivity of human erythrocyte is largely due to the inorganic ions composed primarily of free moving charges such as K^+, Na^+, Mg^{++}, Cl^-, and HCO_3^- and bound charge of haemoglobin. The concentration of ions of haemoglobin is due to its net charge depending on external pH. The value of +45 mmol charges l^{-1} (cell H_2O) was calculated from a total concentration of haemoglobin of 7 mM (cell H_2O) and so the mean effective +ve charge per haemoglobin molecule is +6.4 at the isoelectric point ($pH = pH_{iso} = 6.8$ at 25°C).

Generally, measurements of internal conductivity of human erythrocyte were obtained from cells which were under controlled and well-defined conditions *but not necessarily under realistic physiological conditions*. For example, the osmolality of the suspension medium was kept constant at a value of 300 mOsmol kg^{-1} (Despa, 1995; Gimsa et al., 1994; Sudsiri et al., 2002) and a standard volume (V_{std}) with a value of 90 μm^3 (Richieri et al., 1985). Typically the experimental benchmark temperature was 25 ^{o}C not 37 ^{o}C. However, if the cell is suspended in higher or lower salt concentration, the cells adjust their osmotic pressure by changing their cell volume. Glaser and Donath (1984) introduced the dilution coefficient term (τ) for any volume change (ΔV) from the physiological state and is given as:

$$\tau = \frac{w_c \Delta V}{\Delta V - (1 - w_c) V_{std}} \qquad \text{(Suppl Eq. 5)}$$

where, w_c is the relative cell water content.

In vivo, relative volume, V_{std} of human erythrocyte is defined as 100 % at the standard value for the Mean Cell Volume (MCV) of HRBC (90 μm^3) which is calculated from the routinely measured haematocrit cell volume (H_{ct}). Under such conditions the HRBC possesses a standard relative cell water content (w_{std}) of 0.71 %, and the dilution coefficient (τ) is equal to 1. This benchmark condition is characterized by a standard

intracellular concentration of ions (c_{std} in mM). Therefore, the concentration of non-exchangeable solutes ($c_{non\text{-}exch}$) in erythrocytes with an experimental volume V_c varied by internal ionic changes is given as:

$$c_{non-exch} = \tau c_{std} \qquad \text{(Suppl Eq. 6)}$$

When human erythrocytes are suspended in non-physiological conditions such as high concentration of NaCl or sucrose, water efflux or influx may occur for the cells to balance themselves to the altered osmotic pressure by adjusting cell volume and cell water. Both quantities can be obtained experimentally and consequently the τ can be calculated. The calculation of τ (Suppl Eq. 5) indicates the cytoplasmic concentration of non-exchangeable ions (Suppl Eq. 6) and the cytoplasmic conductivity (Suppl Eq. 1) when Λ_i of each ionic charge in cytoplasm is known and expressed in mol l^{-1} of charge equivalents (Bockris et al. 1973).

However, the cytoplasm is not an ideal solution, in particular, it contains a haemoglobin solution whose Newtonian viscosity is about 5.91 mPa s at 37°C (Cokelet and Meiselman, 1968). The viscosity generates a drag force hindering the ion movements and reduces equivalent conductivity from that of an ideal solution (Suppl Eq. 1). The effect of viscosity (η) on equivalent conductivity of any solution is known as Walden's Law as expressed in Suppl Eq. 7 below (Bockris et al., 1973):

$$\Lambda\eta = \frac{z e_0^- F}{6\pi r} \qquad \text{(Suppl Eq. 7)}$$

where, Λ is the molar conductivity of the ideal solution defined in Suppl Eq. (1), $\Lambda\eta$ is the effective conductivity as a result of viscosity, z is the valence of the ion, e_0^- is the charge on the electron = 1.60217662 x 10^{-19} C, F is the Faraday constant, and r is an effective radius of the ions presented in Bockris et al. (1973) which have a constant value for any solvent solution at a given temperature.

In this work, human erythrocytes were suspended in external media with varying Na^+ concentrations and cytoplasmic conductivities calculated using Suppl Eq. (1), where the value of Λ_i was obtained from Suppl Eqs. (2 and 7). The ionic concentration was

calculated from Suppl Eqs. (5 and 6) where w and V in Suppl Eq. (6) were measured experimentally. The calculated conductivities caused by external Na^+ concentration may influence SAR (Specific Absorption Rate, W kg^{-1}) of human red blood cells (HRBCs) which is defined as:

$$SAR = \frac{dP_V}{dm} = \frac{\sigma_c E^2}{2\rho} \qquad \text{(Suppl Eq. 8)}$$

where, P_V is power absorbed to volume of the tissue (V), dm is the mass of a certain infinitesimal volume (kg), E is the magnitude of internal electric field generated within the tissue in (Volts m^{-1}) and σ_c the conductivity of cytoplasmic HRBC at different external media in S m^{-1}, ρ is HRBC density with a value of 1090 (kg m^{-3}) (Usha et al., 2018).

References

Bockris, J.O., Reddy, I., Amulya, K.N., 1973. Modern electrochemistry, 2nd ed. Plenum Publishing Corporation Press, New York.

Cokelet, G.R., Meiselman, H.J., 1968. Rheological comparison of hemoglobin solutions and erythrocyte suspensions. Science 162, 275–277.

Despa, S., 1995. The influence of membrane permeability for ions on cell behaviour in an electric alternating field. Phys. Med. Boil. 40, 1399–1409.

Gimsa, J., Schnelle, T., Zechel, G., Glaser, R., 1994. Dielectric spectroscopy of human erythrocytes: investigations under the influence of nystatin. Biophys. J. 66, 1244–1253.

Glaser, R., 2000. Biophysics. Springer Press, Berlin.

Glaser, R., Donath, J., 1984. Stationary ionic states in human red blood cells. Bioelectrochem. Bioenerg. 13, 71–83.

Lide, R., 2002. CRC Handbook of Chemistry and Physics. CRC Press, Florida.

Pauly, H., Schwan, H.P., 1966. Dielectric properties and ion mobility in erythrocyte. Biophys J 6:, 621–639.

Richieri, G. V, Akeson, S.P., Mel, H.C., 1985. Measurement of biophysical properties of red blood cells by resistive pulse spectroscopy: volume shape, surface area, and deformability. J. Biochem. Biophys. Methods 11, 117–131.

Sudsiri, C.J., Ritchie, R.J., 2021. Energy absorption of human red blood cells and conductivity of the cytoplasm influenced by temperature. Biophys. Chem. 273, 106578.

Sudsiri, J., Wachner, D., Donath, J., Gimsa, J., 2002. Can molecular properties of human red blood cells be accessed by electrorotation? Songklanakarin J. Sci. Technol. 24, 785–789.
